# Monolithic mode-selective *few-mode multicore* fiber multiplexers

**DOI:** 10.1038/s41598-017-06561-w

**Published:** 2017-08-01

**Authors:** Nicolas Riesen, Simon Gross, John D. Love, Yusuke Sasaki, Michael J. Withford

**Affiliations:** 1 The Institute for Photonics and Advanced Sensing (IPAS) and School of Physical Sciences, The University of Adelaide, Adelaide, Australia; 2 School of Engineering, University of South Australia, Mawson Lakes, Australia; 3 Centre for Ultrahigh bandwidth Devices for Optical Systems (CUDOS), MQ Photonics Research Centre, Department of Physics and Astronomy, Macquarie University, Sydney, Australia; 4 Research School of Physics and Engineering (RSPE), The Australian National University, Canberra, Australia; 5 Advanced Technology Laboratory, Fujikura Ltd. 1440, Mutsuzaki, Sakura, Chiba Japan

## Abstract

With the capacity limits of standard single-mode optical fiber fast approaching, new technologies such as space-division multiplexing are required to avoid an Internet capacity crunch. Few-mode multicore fiber (FM-MCF) could allow for a two orders of magnitude increase in capacity by using the individual spatial modes in the different cores as unique data channels. We report the realization of a monolithic mode-selective few-mode multicore fiber multiplexer capable of addressing the individual modes of such a fiber. These compact multiplexers operate across the S + C + L telecommunications bands and were inscribed into a photonic chip using ultrafast laser inscription. They allow for the simultaneous multiplexing of the LP_01_, LP_11a_ and LP_11b_ modes of all cores in a 3-mode, 4-core fiber with excellent mode extinction ratios and low insertion losses. The devices are scalable to more modes and cores and therefore could represent an enabling technology for practical ultra-high capacity dense space-division multiplexing.

## Introduction

In recent years techniques such as dense wavelength-division multiplexing (DWDM), polarization multiplexing, multilevel modulation, coherent detection and advanced digital signal processing have allowed the data capacity of standard single-mode optical fiber (SMF) to reach 100 Tb/s^1, 2^. However, with the exponential increase in Internet data demand, new technologies are required to further increase optical fiber capacity to avoid an impending capacity crunch^1–3^. Space-division multiplexing (SDM) is the most promising solution, and it involves the addition of extra data channels to the cross-section of an optical fiber^1^. SDM entails the use of either multicore fiber (MCF) in which multiple single-mode fiber cores are placed into a common cladding, or multimode fibers (MMF) in which the different transverse modes (or mode groups) supported by the fiber are used as individual data channels in an approach referred to as mode-division multiplexing (MDM)^4–6^. SDM has the potential to provide orders of magnitude improvements in the spectral efficiency and also lower energy requirements, reducing the cost per transmitted bit when compared with the linear scaling of capacity by merely using more SMFs in parallel^1, 7^. Recently the number of cores in MCF demonstrations has increased beyond 20, allowing for >2 Pb/s data transmission^8^, whereas mode-multiplexed MMF networks using up to 15 linearly-polarized (LP) modes have been reported^9^. To potentially allow for even greater increases in fiber capacity, focus has recently turned to combining the two approaches by using few-mode multicore fiber (FM-MCF)^10–14^. FM-MCF is widely believed to provide the most practical balance between high-order uncoupled SDM and low-order MDM. Since FM-MCF allows for the number of spatial channels to be scaled through increases in both the core and mode multiplicity, dramatic improvements in fiber capacity of up to 2 orders of magnitude are predicted^15^. To date FM-MCFs with 10’s to 100’s of spatial channels have been realized^13, 16, 17^, with a recent demonstration of a 6-mode, 19-core fiber allowing for data rates as high as 2.05 Pb/s^16^. A FM-MCF with 36 spatial channels was also recently demonstrated for data transmission over 500 km^11^.

Whilst FM-MCFs promise significant increases in fiber capacity, practical methods of exciting the individual modes supported by the closely-spaced cores are still required for the eventual deployment of FM-MCF networks. Most FM-MCF demonstrations to date have however relied on elaborate, often impractical and typically lossy free-space spatial multiplexers which in some cases require the individual cores to be addressed one after the other^13, 18–21^. For these reasons simple waveguide-based technologies with the potential for low loss and small footprints, have been sought to make SDM of FM-MCF more practical.

Since the multiplexing of higher-order modes in a FM-MCF is naturally suited to three-dimensional waveguide architectures, we use ultrafast 3D laser inscription (ULI) for the multiplexer fabrication^3, 22–27^. We report the successful integration of an array of mode-selective couplers and a fan-in device into a single compact monolithic photonic chip^28^. Each individual tapered mode coupler allows for direct one-to-one mode multiplexing of the LP_01_, LP_11a_ and LP_11b_ modes of a given fiber core across the entire S + C + L bands.

The integrated FM-MCF multiplexer^28^ represents a significant advancement over previous work in which ULI was used to fabricate tapered mode couplers operating in the visible^26^, and non-tapered mode couplers operating within the C-band^27^. The results also relate to the realization of fan-in/fan-out devices, spot-based FM-MCF couplers and photonic lanterns using the same fabrication approach^3, 22–25^. The devices presented are also closely related to an all-fiber dissimilar-core photonic lantern recently fabricated for FM-MCF (3 modes × 7 cores) but for which only preliminary results were given^29^.

Importantly, the one-to-one mode mapping functionality of the devices presented here could allow for the compensation of differential mode delay (DMD) and mode-dependent loss (MDL) in coherent SDM networks. Alternatively, the spatial multiplexers could be used for SDM in time-division multiplexed (TDM) passive optical networks (PON) without the requirement for sophisticated digital signal processing (DSP) when using low-crosstalk FM-MCF^30^.

The spatial multiplexers reported exhibit excellent mode purity and the lowest overall insertion losses of any few-mode multicore fiber multiplexer reported to date. Moreover, the fabrication technique offers scalability to more modes and cores and is also suited to mass-production^24, 28^.

## Results

### Adiabatic mode-selective coupling

**Figure 1:**
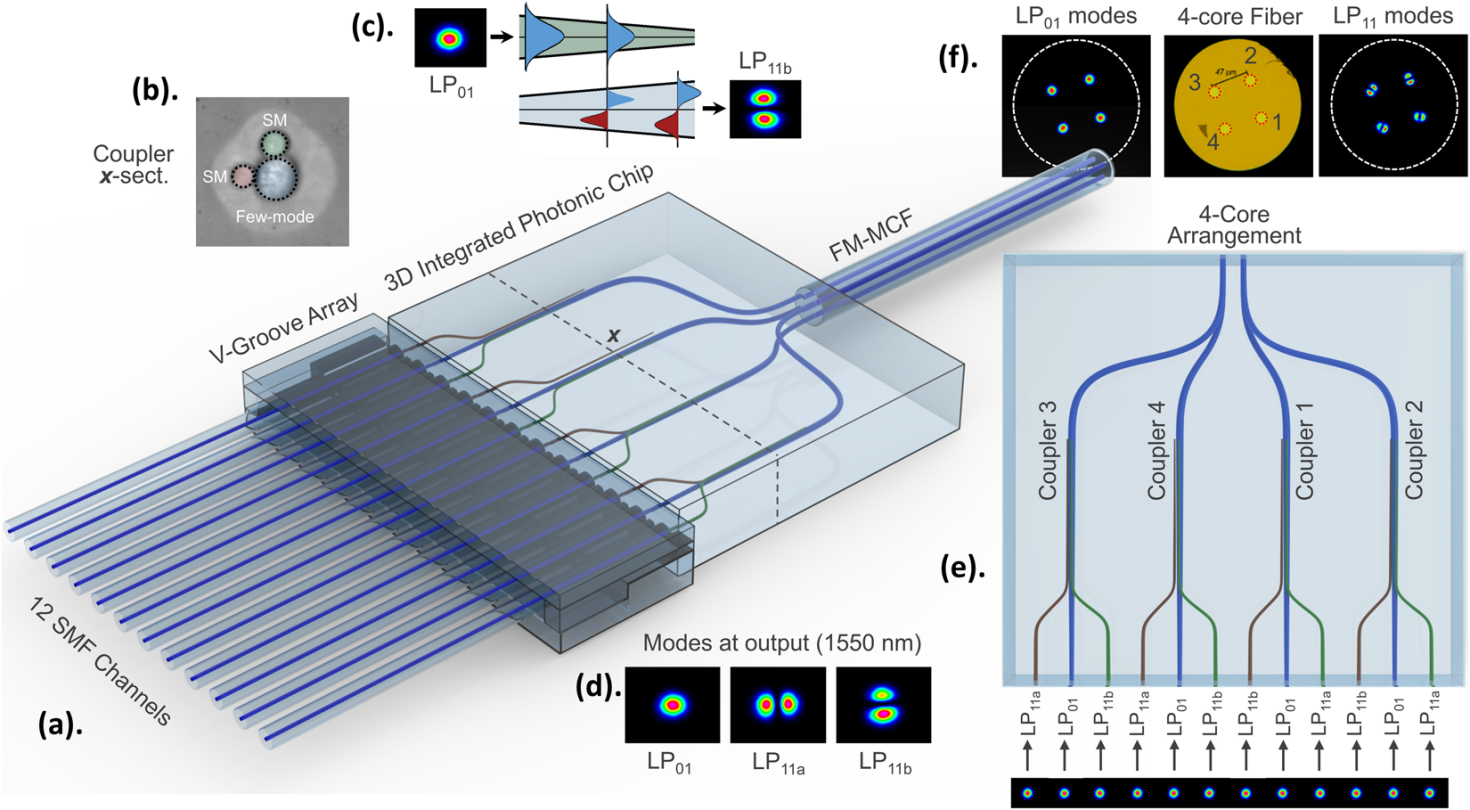
Fabricated integrated FM-MCF multiplexer. (**a**) The FM-MCF multiplexer consists of 4, 3-core tapered mode couplers. (**b**) Each 3-core tapered coupler consists of an up-tapered few-mode waveguide (highlighted in blue) surrounded by two down-tapered single-mode waveguides (highlighted in red and green) with a 90 degree angular offset to allow for multiplexing both the LP_11a_ and LP_11b_ modes in the few-mode waveguide. Shown is a microscope image of one coupler’s cross-section at position *x* (prior to thermal annealing). With post-annealing of the glass the large outer index modification (light grey region) is erased^44^. The adiabatic mode evolution in such a coupler is demonstrated in (**c**) and the LP_01_, LP_11a_ and LP_11b_ modes of one of the cores imaged using an infrared camera are shown in (**d**). After the coupling region the few-mode waveguides (**e**) fan-in to interface with the (**f**) 3-mode, 4-core fiber allowing each individual mode to be excited independently.

The integrated FM-MCF multiplexers reported in this paper^28^ were designed to simultaneously multiplex all the LP_01_, LP_11a_ and LP_11b_ modes supported in each of the cores of the 4-core fiber^31^ shown in Fig. 1(f). The FM-MCF spatial multiplexer is shown in the schematic of Fig. 1(a) and consists of an array of four tapered mode couplers that are positioned side-by-side^28^. Tapered mode couplers involve an adiabatic evolution of the fundamental mode in one waveguide to a higher-order mode in an adjacent waveguide. They were chosen because they are ultra-broadband in performance and exhibit high fabrication tolerances. This is because, unlike standard directional mode couplers, they do not have a prescribed coupling length and also do not require precise phase-matching conditions to be satisfied over a prolonged distance^26, 32^.

The individual tapered mode couplers, Fig. 1(b), have three cores (i.e. 1 few-mode waveguide surrounded by 2 single-mode waveguides with 90° angular offset)^33, 34^. The single- and few-mode waveguides are counter-tapered and of different diameters ensuring that the propagation constant of the fundamental modes in the single-mode waveguides match those of the LP_11_ modes of the larger few-mode waveguide somewhere in the middle of the taper^32^. This enables the horizontal single-mode waveguide to couple with the LP_11a_ mode of the few-mode waveguide, whereas the vertical waveguide having a 90 degree angular offset couples to the orthogonal LP_11b_ mode as shown in Fig. 1(c),(d) ^26, 33^. S-bends at the input and output of the tapered mode couplers spatially separate the waveguides. The bend radii for the single-mode and few-mode waveguides were kept larger than 75 mm and 90 mm, respectively, to ensure negligible bend losses. To ensure negligible intercore crosstalk the separation between the waveguides was >45 µm. The few-mode waveguides from the four couplers are then routed in such a way that they interface with the four cores of the fiber as shown in Fig. 1(e). Towards the input of the device all 12 waveguides are tapered down to the same (single-mode) diameter, forming a linear arrangement, to allow interfacing with a V-groove fiber array with 127 µm pitch. The overall length of the ULI written device is 40 mm, with the interaction length of the couplers being 15 mm. The FM-MCF multiplexer was initially designed using Finite Element and Beam Propagation Method simulations. The geometry and laser inscription parameters were then further optimized experimentally to achieve high mode purity, low insertion losses and a broad operational bandwidth.

### Chip Characterization

**Figure 2:**
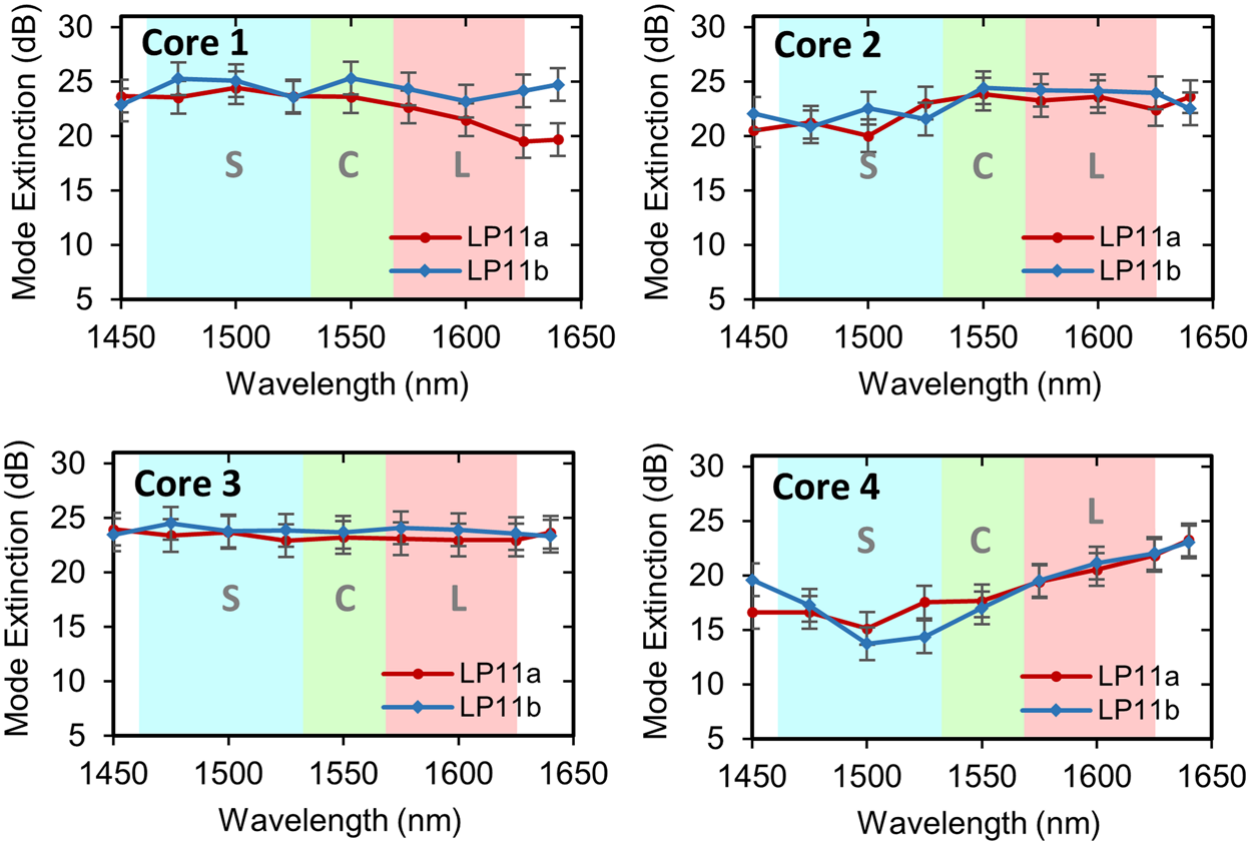
Chip mode extinction ratios. Shown are the mode extinction ratios (power ratio between LP_01_ and LP_11_ modes) for each of the four tapered mode couplers in the FM-MCF spatial multiplexer chip.

**Figure 3:**
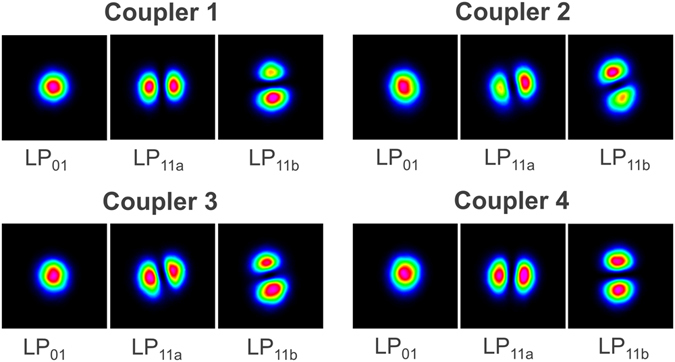
Mode intensity profiles. The LP_01_, LP_11a_ and LP_11b_ modes at 1550 nm imaged at the output ports of the FM-MCF multiplexer.

The mode-selectivity and mode purity of the FM-MCF multiplexer was quantified by the mode extinction ratios (MER). The MERs for the four tapered couplers within the FM-MCF multiplexer chip are shown in Fig. 2 for a subsection of the operational bandwidth. For launching into the LP_11a_ and LP_11b_ modes, the device exhibits between 14–25 dB mode extinction across the entire S + C + L bands, highlighting the broadband performance of the device. The average mode extinction ratio for all modes and cores was 22 dB. Note that the lower MERs at shorter wavelengths observed for core 4 are likely due to a fabrication imperfection for that particular coupler. These MERs compare favorably with single-core few-mode fiber mode multiplexers reported in the literature (>6 dB^35^, >15 dB^36^, ~ 21 dB^37^). The LP_01_, LP_11a_ and LP_11b_ mode profiles of the output cores of the FM-MCF multiplexer at 1550 nm are shown in Fig. 3. This figure showcases the high mode purity and symmetry realized using these ultra-broadband mode couplers.

**Figure 4:**
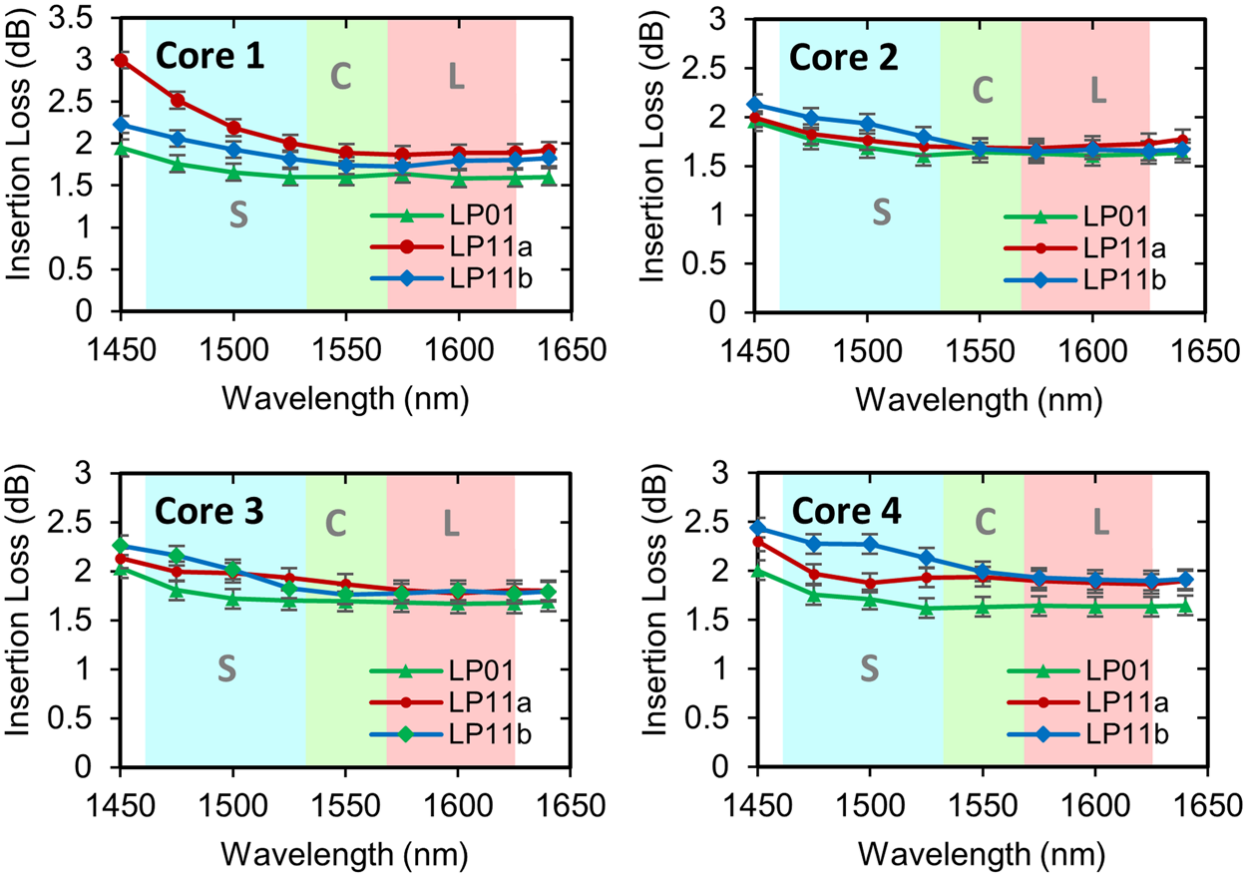
Chip insertion losses. Shown are the insertion losses for each of the four tapered mode couplers in the FM-MCF spatial multiplexer chip.

As shown in Fig. 4, the insertion losses (IL) ranged between 1.6–3.0 dB over the S + C + L bands with an average of 1.8 dB, for all modes and cores. This compares favorably to other few-mode multicore multiplexers reported to date (<5 dB^10^, ~ 6 dB^3, 16^, 6.3–9.4 dB^13^). The insertion losses are predominately due to Fe impurities within the glass, and could in principle be reduced to well below 1 dB with the use of ultra-high-purity glass^38^. The impurities contribute approximately 0.27 dB/cm loss to the overall propagation losses of 0.32 ± 0.05 dB/cm for straight single-mode waveguides at 1550 nm^38^.

The LP_11_ modes in general have slightly higher insertion losses than the LP_01_ mode, by approximately 0.2 dB on average, because of the extra bend losses from the couplers. Note also that the slight increase in losses towards shorter wavelengths for all four cores is due to the tail of the OH absorption in the glass (i.e. the OH band is centered at 1400 nm) as previously reported^38^.

The addition of a V-groove fiber array increased the insertion losses by an average of 0.2 dB due to slight misalignments arising from the dimensional tolerances of the array. No polarization dependency was observed for the insertion losses and mode extinction ratios.

### 4-core fiber interface

**Figure 5:**
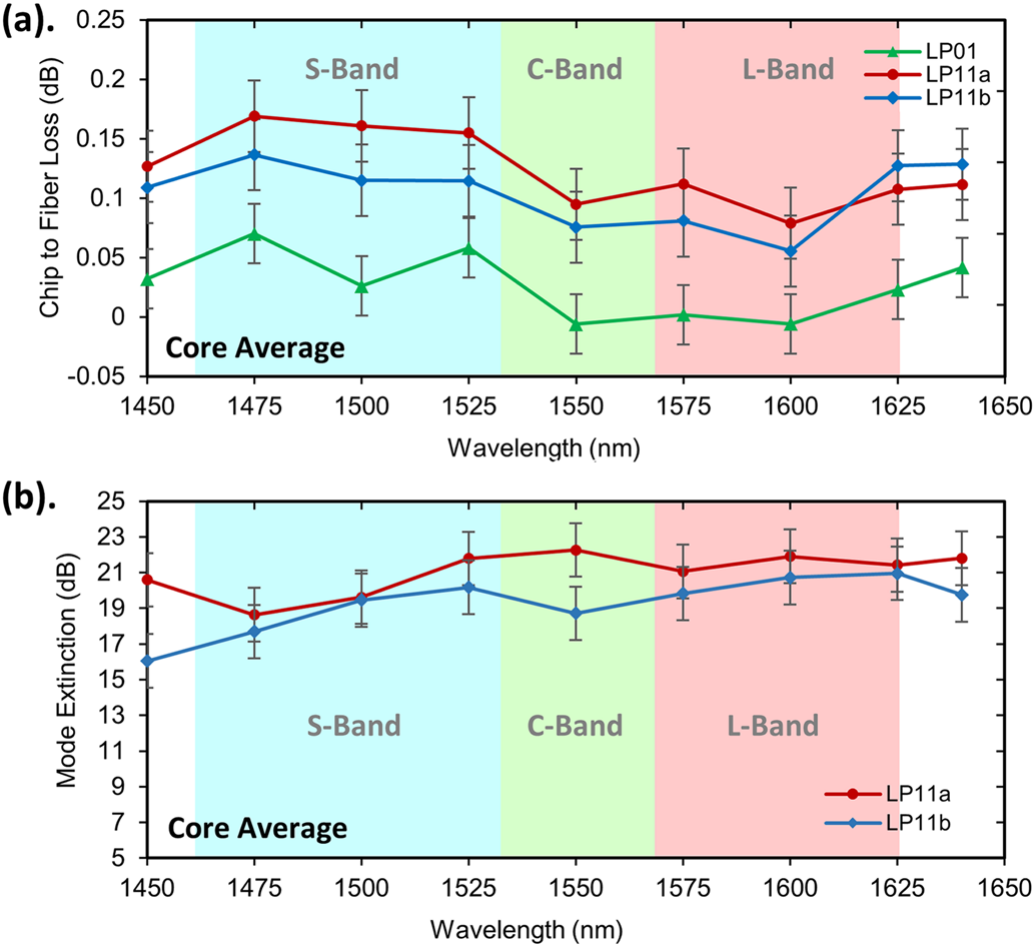
Chip to FM-MCF interface performance. (**a**) The coupling losses between the FM-MCF multiplexer and the 4-core fiber butt-coupled to the end-face of the chip. (**b**) The mode extinction ratios for the FM-MCF multiplexer after a 10 m length of the 4-core fiber butt-coupled to the end-face of the chip. The values are given as averages across all four cores.

A 10 m length of the 3-mode, 4-core fiber was then butt-coupled to the end-face of the glass chip with alignment ensured using fine angular adjustment. The increase in insertion losses was then used to infer the chip-to-fiber coupling losses which, as shown in Fig. 5(a), were less than 0.18 dB, with an average of just 0.1 dB, across the S + C + L bands. The low losses can be attributed to well-matched chip and fiber mode-profiles. The Fresnel reflection at the interface between the Eagle2000 glass chip (1.488 at 1550 nm) and the fiber (1.444 at 1550 nm) is calculated to be as low as 0.02% when using adhesive index-matched to either the fiber or chip. Furthermore, back reflections could be eliminated almost entirely by polishing the chip and fiber at angles. The mode extinction ratios remained almost unchanged after interfacing with the FM-MCF and ranged from 13–25 dB with an average of 20 dB, which is just 2 dB lower than the chip. The mode extinction ratios (16–22 dB) for the LP_11a_ and LP_11b_ modes, averaged across the 4 cores, are shown in Fig. 5(b). Note that whilst the LP_11a_ and LP_11b_ modes exiting the chip are orthogonal and uncoupled due to the slight asymmetry of the ULI waveguides which lifts the degeneracy, coupling between the modes inevitably occurs along the length of the abutted FM-MCF causing mode rotation. Depending on the power ratio between the two LP_11_ orientation-states a ring-like intensity profile can be observed at the outputs of the FM-MCF as would be expected.

**Figure 6:**
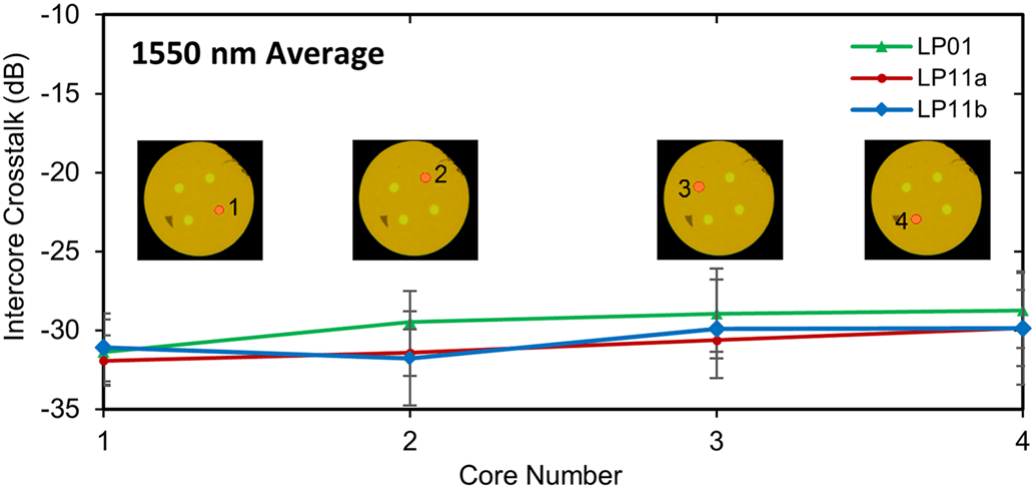
Intercore crosstalk. The intercore crosstalk at the end of the 4-core fiber, when exciting the modes with the FM-MCF spatial multiplexer at 1550 nm. The intercore crosstalk when exciting a given LP_*lm*_ mode in a given core, is calculated from the average crosstalk between the given core and each of other cores.

The intercore crosstalk was also characterized as shown in Fig. 6, by measuring the output power of each core of the FM-MCF as each single-mode input was individually excited. The overall intercore crosstalk arising from the multiplexer chip, the chip-to-fiber interface and the short length of fiber was less than −28 dB as shown in Fig. 6 over the entire wavelength range for all 12 modes. Whilst the intercore crosstalk for the LP_11_ mode is generally higher than for the LP_01_ mode in FM-MCFs^31^, the opposite appears here, although it must be said that the discrepancy is within the uncertainty of the results. With only a short length of fiber used the intercore crosstalk is dominated instead by the chip-to-fiber interface. This contribution arising from imperfect alignment and mode-mismatch is believed to dominate over scattering caused by the bends and small irregularities of the waveguides inside the chip. It is therefore expected that the intercore crosstalk could be reduced to below −40 dB, as required for long-haul applications, with further optimization of the alignment and the ULI parameters. In particular, improved mode-matching between the chip and the FM-MCF and SMF would be critical. Apart from further optimization of the inscription parameters, mode-matching could be improved using graded-index fiber, or lower index glass for the chip. Shallower bends could also be used to reduce light leakage.

Low intercore crosstalk levels are particularly beneficial in intermediate- to long-haul fiber networks, improving the transmission length and achievable capacity, whilst mitigating the need for further digital signal processing^13^. The intercore crosstalk levels achieved in the present multiplexing demonstration are commensurate with QAM and QPSK transmission over tens to hundreds of kilometers of the fiber with relatively low BERs^39^.

### Fiber array interface

**Figure 7:**
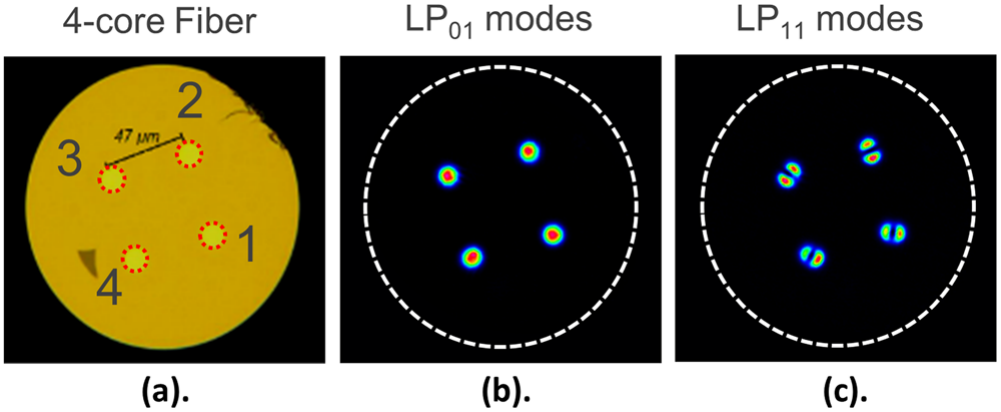
Simultaneous mode multiplexing. (**a**) The 3-mode, 4-core fiber, with the simultaneous multiplexing of all the (**b**) LP_01_ and (**c**) LP_11_ modes shown at 1550 nm.

For the previous results each individual mode was addressed in succession, with the alignment of the input fiber re-adjusted each time. To allow all 12 spatial channels to be multiplexed simultaneously the 12 single-mode inputs of the multiplexer have to be interfaced with a 127 micron pitch fiber array. The multiplexing of all LP_01_ and LP_11_ modes in the cores using such a fiber array is shown in Fig. 7. In this case the alignment is more challenging since several waveguides must be optimized simultaneously. For this reason, a slight compromise in the mode extinction ratios and insertion losses could occur due to micron-scale misalignments. In the present case the mode extinction ratios remained almost unchanged having an average of 20 dB across the S + C + L bands. The insertion losses, however, were approximately 0.2 dB higher. The performance of the FM-MCF spatial multiplexer therefore remains very high even when using a V-groove fiber array for simultaneously multiplexing all 12 modal channels.

## Methods

### 3-mode, 4-core fiber

The most basic FM-MCFs, such as the one used in this paper, have cores with step-index refractive index profiles, which however limits the core number to about seven due to the higher intercore crosstalk. Increasing the core number by reducing the core-to-core pitch would require more sophisticated core profiles^12, 15, 40–43^. Few-mode heterogeneous FM-MCFs with air hole-assisted double cladding structures could for instance allow for as many as 387 spatial channels^15^. The specific FM-MCF used here was fabricated using a stack and draw method and is identical to Fiber C in Ref 31. The fiber has a spatial channel count of 12 and the cores have a step-index refractive index profile, a radius *ρ* = 6.89 µm and a relative index difference of Δ = 0.45%. The design of the FM-MCF required a careful trade-off between multiple key parameters including intercore crosstalk, fiber nonlinearity (or mode area) and core density^31^.

### 3-mode, 4-core multiplexer fabrication

The FM-MCF multiplexers were fabricated using a high repetition rate Ti:Sapphire oscillator (Femtolasers FEMTOSOURCE XL500, 800 nm, 5.1 MHz, <50 fs). The circularly polarized laser beam was focused ~200 μm below the top surface of a boro-aluminosilicate glass sample (Corning Eagle2000, *n*  ~ 1.49 at 1550 nm) with a 100 × 1.4 NA oil immersion microscope objective (Olympus UPLANSAPO). The sample was translated using high precision XYZ air-bearing stages (Aerotech) and maneuvered at a constant speed of 250 mm/min. Tapering of the waveguides was realized by linearly changing the pulse energy with a computer controlled attenuator (Aerotech ADR75 rotation stage and a half-wave plate). The waveguide diameters (and corresponding laser pulse energies) were chosen based on numerical simulations and then further optimized experimentally. The single-mode waveguides were written using a pulse energy of 124 nJ and linearly down-tapered in the coupling region to a pulse energy of 104 nJ towards the output of the device. The few-mode waveguides interfacing with the 4-core fiber were inscribed with a pulse energy of 217 nJ to create waveguides of size ~21 × 24 µm (H × V). We note that the slight asymmetry of the waveguides has minimal influence on the mode symmetry or purity. In the coupling region the few-mode waveguides were counter-tapered, relative to the single-mode waveguides, down to 180 nJ. The waveguides are then tapered down to 104 nJ to make them single-mode towards the input of the device. The interaction region for the couplers was 15 mm long with an axis-to-axis waveguide separation in the middle of the interaction region of ~10 µm and ~12 µm for the horizontal and vertical single-mode waveguides, respectively. To avoid shadowing effects the deepest waveguides were written first and the device was built upwards towards the top surface. After all four 3-core couplers and the fan-in waveguide configuration were inscribed, the glass chip was post-annealed in order to simplify the waveguide refractive index profiles, resulting in waveguides with a positive index change surrounded by a depressed ring^26, 44^. The peak positive refractive index contrasts were measured to be 0.40% at the lowest pulse energy of 104 nJ and 0.53% at the largest pulse energy of 214 nJ using a refracted near-field profilometer (Rinck Elektronik) at 635 nm.

### 3-mode, 4-core multiplexer characterization

The 3-mode, 4-core spatial multiplexers were characterized by launching light into each single-mode input individually and imaging the end-face of the glass chip using an infrared camera (FLIR SC7000) 150 mm away from a 50× LMPLAN-N objective. This infrared camera was chosen specifically for its large dynamic range and high linearity. Any nonlinearity was deemed to have a negligible impact on the uncertainty of the measurements, which were dominated by the background noise. The light (1–10 mW) was launched via butt-coupling to the chip a single-mode fiber (Corning SMF-28e) connected to a Yenista Tunics T100S-HP tunable laser. The mode extinction ratios (defined as the power ratio of LP_01_ and LP_11_ modes) were calculated based on the near-field profiles^27, 45^, with the null of the LP_11_ mode in the few-mode waveguide providing an estimate of the LP_01_ contamination. Whilst mode extinction ratios are ideally calculated from two back-to-back devices, the use of the near-field pattern from one device is adequate in this case given that only two modes are supported in the waveguides. This implies that any signal in the null of the LP_11_ mode can only be due to LP_01_ mode contamination. The intensity of the LP_01_ mode content in the LP_11_ mode is then determined by scaling the null level by the intensity (integrated)-to-peak calibration obtained from the LP_01_ mode launch as described previously^45^. The null of the LP_11_ mode was determined from a 17 × 17 pixel window, after subtracting the mean background and applying a 2 × 2 moving average filter. The total power was integrated over a 120 pixel diameter circle centered on the mode. We note however that this measurement technique would not be suitable when more mode families are present, in which case it is imperative to measure the transfer matrix of the system. The insertion losses were determined by imaging the injection fiber and chip outputs onto an optical power meter (Thorlabs S146C), using an aspheric lens, and comparing the measured power values. The injection alignment was optimized at 1550 nm for each port. The intercore crosstalk when exciting a given LP_*lm*_ mode in a given core, was calculated from the average crosstalk between the given core and each of other cores. This was determined from all the power measurements of the individual output cores when separately exciting each of the inputs at 1550 nm.

## Discussion

Spatial multiplexers including those presented in this paper, often map the single-mode inputs directly to individual modes within a fiber core. We note however that one-to-one mode mapping is not actually essential in coherent SDM networks. Spot-based couplers and photonic lanterns which are often used in coherent networks, typically scramble the single-mode inputs across an orthogonal combination of all mode channels in a particular core of the fiber^22^. MIMO digital signal processing (DSP) then allows for the individual orthogonal modal channels to be recovered at the end of the fiber^22, 23, 46^. Moreover, it is not necessary to demultiplex each mode within a given mode group as the nearly-degenerate modes will have already undergone significant cross-coupling along the fiber^35, 46^. In practice however there are very significant benefits to one-to-one mode mapping in terms of mode-dependent loss (MDL) and differential mode delay (DMD) compensation^29, 47^. In coherent SDM networks the MIMO DSP depends very strongly on both the MDL and the DMD. MDL reduces the system capacity and increases the outage probability^48^. MIMO DSP can only completely recover the modal data channels by inverting the transfer matrix if the scrambling is unitary which is to say the MDL is almost zero^22^. Therefore the one-to-one mode mapping capability is highly beneficial in terms of equalizing the MDL by allowing for the mode launch power or the mode gain to be varied^49^. The latter could be achieved for instance by varying the modal pump power using a variant of the mode multiplexer demonstrated in this paper that operates at 980 nm. Furthermore, one-to-one mode mapping multiplexers allow for the compensation of DMD without requiring long lengths of intermodal dispersion compensating fibers or low DMD fibers. DMD determines the number of taps required for intra-core (and/or intercore) crosstalk equalization^3^, and thus DMD compensation is necessary to avoid inhibitively high DSP complexity and to minimize energy requirements^50^. For long-haul systems graded-index fibers are generally used for minimizing DMD and MDL. The fabrication technique reported here is suitable for interfacing with such graded-index fiber^27, 51^. One-to-one mode mapping multiplexers could also be used for SDM transmission in fibers with low overall crosstalk using direct detection, without requiring MIMO DSP^30^. The FM-MCF multiplexers demonstrated in this paper are therefore a significant development in the pursuit of practical space-division multiplexing for overcoming the optical fiber capacity crunch. As suggested, the technology could also be readily adapted for the mode-selective pumping of few-mode multicore EDFAs for modal gain control^19, 52^ potentially allowing for a practical solution to yet another key component of SDM networks.

In summary, we have demonstrated a one-to-one mode mapping FM-MCF multiplexer^28^ fabricated using ultrafast laser inscription. We have also provided the first demonstration of a multicore mode-selective multiplexer coupled to a FM-MCF. The FM-MCF multiplexer allows for the multiplexing of the LP_01_, LP_11a_ and LP_11b_ modes of each individual core of a 4-core fiber over a bandwidth exceeding the S + C + L bands. The mode-selective functionality is achieved using a tapered mode coupler array integrated with a fan-in architecture. The mode extinction ratios, with a 10 m length of the 3-mode, 4-core fiber butt-coupled to the end-face of the multiplexer chip, ranged from 13–25 dB with an average of 20 dB over the operational bandwidth, whilst the insertion loses were only 1.8 dB on average. The intercore crosstalk from the multiplexer was found to be less than −28 dB, which could be reduced even more with further refinement of the ULI parameters. Compared to photonic lanterns, the FM-MCF multiplexers presented here have greatly improved mode-selectivity but higher insertion losses^22, 46^. The FM-MCF multiplexer’s small footprint could allow for integration into future transponders for both coherent and direct detection networks. In coherent networks the mode-selective functionality would allow for MDL and DMD compensation, with the latter significantly reducing the MIMO DSP complexity. The FM-MCF multiplexers could also be used in time-division multiplexed (TDM) passive optical networks (PON) without requiring MIMO DSP^30^. Moreover, the versatility of the fabrication technique used could allow the multiplexing capability to be scaled to many more cores and modes. The ultrafast laser inscription technique used also has high repeatability and lends itself towards volume-scalable production. Given the dramatic increases in optical fiber capacity that may be possible with FM-MCFs, this practical and scalable approach to multiplexing the cores represents a significant step forwards towards the realization of practical DSDM.

## References

[1] Richardson D, Fini J, Nelson L (2013). Space-division multiplexing in optical fibres. Nat. Photon..

[2] Li G, Bai N, Zhao N, Xia C (2014). Space-division multiplexing: the next frontier in optical communication. Adv. Opt. Photonics.

[3] Van Uden R (2014). Ultra-high-density spatial division multiplexing with a few-mode multicore fibre. Nat. Photon..

[4] Mizuno T, Takara H, Shibahara K, Sano A, Miyamoto Y (2016). Dense space division multiplexed transmission over multicore and multimode fiber for long-haul transport systems. J. Lightw. Technol..

[5] Saitoh, K. Multicore fiber technology. *OFC*, paper Th4C.1, doi:10.1364/OFC.2015.Th4C.1 (2015).

[6] Luo LW (2014). WDM-compatible mode-division multiplexing on a silicon chip. Nat. Comm..

[7] Kahn JM, Miller DA (2017). Communications expands its space. Nat. Photon..

[8] Puttnam, B. *et al*. 2.15 Pb/s transmission using a 22 core homogeneous single-mode multi-core fiber and wideband optical comb. *ECOC*, paper PDP.3.1, doi:10.1109/ECOC.2015.7341685 (2015).

[9] Fontaine, N. K. *et al*. 30 × 30 MIMO transmission over 15 spatial modes. *OFC*, paper Th5C.1, doi:10.1364/OFC.2015.Th5C.1 (2015).

[10] Mizuno, T. *et al*. 12-core × 3-mode dense space division multiplexed transmission over 40 km employing multi-carrier signals with parallel MIMO equalization. *OFC*, paper Th5B.2, doi:10.1364/OFC.2014.Th5B.2 (2014).

[11] Shibahara, K. *et al*. Dense SDM (12-core × 3-mode) transmission over 527 km with 33.2-ns mode-dispersion employing low-complexity parallel MIMO frequency-domain equalization. *OFC*, paper Th5C.3, doi:10.1364/OFC.2015.Th5C.3 (2015).

[12] Tu J, Saitoh K, Takenaga K, Matsuo S (2014). Heterogeneous trench-assisted few-mode multi-core fiber with low differential mode delay. Opt. Express.

[13] Sakaguchi J (2016). Large spatial channel (36-core × 3 mode) heterogeneous few-mode multi-core fiber. J. Lightw. Technol..

[14] Sasaki Y (2015). Few-mode multicore fiber with 36 spatial modes (three modes (LP_01_, LP_11a_, LP_11b_) × 12 cores). J. Lightw. Technol..

[15] Watanabe T, Kokubun Y (2014). Ultra-large number of transmission channels in space division multiplexing using few-mode multi-core fiber with optimized air-hole-assisted double-cladding structure. Opt. Express.

[16] Soma, D. *et al*. 2.05 Peta-bit/s super-nyquist-WDM SDM transmission using 9.8-km 6-mode 19-core fiber in full C band. *ECOC*, paper PDP.3.2, doi:10.1109/ECOC.2015.7341686 (2015).

[17] Sakamoto, T. *et al*. Few-mode multi-core fibre with highest core multiplicity factor. *ECOC*, paper We.1.4.3, doi:10.1109/ECOC.2015.7341964 (2015).

[18] Qian, D. *et al*. 1.05 Pb/s transmission with 109b/s/Hz spectral efficiency using hybrid single-and few-mode cores. *FiO*, paper FW6C.3, doi:10.1364/FIO.2012.FW6C.3 (2012).

[19] Tsuchida, Y., Matsuura, H., Tadakuma, M. & Sugizaki, R. Multicore EDFA for space division multiplexing by utilizing cladding-pumped technology. *OFC*, paper Tu2D.1, doi:10.1364/OFC.2014.Tu2D.1 (2014).

[20] Sakaguchi, J. *et al*. Large-scale, heterogeneous, few-mode multi-core fiber technologies with over 100 spatial channels. *Photonics Conference*, paper MJ2.2, doi:10.1109/IPCon.2015.7323729 (2015).

[21] Sakaguchi, J. *et al*. Realizing a 36-core, 3-mode fiber with 108 spatial channels. *OFC*, paper Th5C.2, doi:10.1364/OFC.2015.Th5C.2 (2015).

[22] Fontaine, N. K. & Ryf, R. Characterization of mode-dependent loss of laser inscribed photonic lanterns for space division multiplexing systems. *OECC*, paper MR2.2 (2013)

[23] Mitchell, P., Brown, G., Thomson, R. R., Psaila, N. & Kar, A. 57 channel (19 × 3) spatial multiplexer fabricated using direct laser inscription. *OF*C, paper M3K.5, doi:10.1364/OFC.2014.M3K.5 (2014).

[24] Thomson R, Birks TA, Leon-Saval S, Kar A, Bland-Hawthorn J (2011). Ultrafast laser inscription of an integrated photonic lantern. Opt. Express.

[25] Guan, B., Ercan, B., Fontaine, N. K., Scott, R. P. & Yoo, S. Mode-group-selective photonic lantern based on integrated 3D devices fabricated by ultrafast laser inscription. *OFC*, paper W2A.16, doi:10.1364/OFC.2015.W2A.16 (2015).

[26] Gross S, Riesen N, Love JD, Withford MJ (2014). Three‐dimensional ultra‐broadband integrated tapered mode multiplexers. Las. Phot. Rev..

[27] Riesen N, Gross S, Love JD, Withford MJ (2014). Femtosecond direct-written integrated mode couplers. Opt. Express.

[28] Riesen, N., Gross, S., Love, J. D., Sasaki, Y. & Withford, M. J. Femtosecond laser written integrated spatial multiplexers for few-mode multicore fibre. *ECOC*, paper P2.SC2.16 (2016).

[29] Antonio-Lopez, E. *et al*. Few mode multicore photonic lantern. *OFC*, paper Tu3l.5, doi:10.1364/OFC.2016.Tu3l.5 (2016).

[30] Xia, C. *et al*. Demonstration of world’s first few-mode GPON. *ECOC, paper PD.1.5*, doi:10.1109/ECOC.2014.6964260 (2014).

[31] Sasaki Y (2012). Large-effective-area uncoupled few-mode multi-core fiber. Opt. Express.

[32] Riesen N, Love JD (2013). Tapered velocity mode-selective couplers. J. Lightw. Technol..

[33] Love JD, Riesen N (2012). Mode-selective couplers for few-mode optical fiber networks. Opt. Lett..

[34] Riesen N, Love JD, Arkwright JW (2012). Few-core spatial-mode multiplexers/demultiplexers based on evanescent coupling. IEEE Phot. Technol. Lett..

[35] Leon-Saval SG (2014). Mode-selective photonic lanterns for space-division multiplexing. Opt. Express.

[36] Hanzawa N (2013). Two-mode PLC-based mode multi/demultiplexer for mode and wavelength division multiplexed transmission. Opt. Express.

[37] Labroille, G., Jian, P., Barré, N., Denolle, B. & Morizur, J.F. Mode selective 10-mode multiplexer based on multi-plane light conversion. *OFC*, paper Th3E.5, doi:10.1364/OFC.2016.Th3E.5 (2016). 10.1364/OE.22.015599 24977818

[38] Meany T (2014). Towards low-loss lightwave circuits for non-classical optics at 800 and 1,550 nm. Appl. Phys. A.

[39] Puttnam B (2016). Impact of intercore crosstalk on the transmission distance of QAM formats in multicore fibers. IEEE Phot. J..

[40] Saitoh K, Matsuo S (2013). Multicore fibers for large capacity transmission. Nanophot..

[41] Tu, J., Saitoh, K., Amma, Y., Takenaga, K. & Matsuo, S. Design method of heterogeneous trench-assisted graded-index few-mode multi-core fiber with low differential mode delay. *OECC*, doi:10.1109/OECC.2015.7340200 (2015). 10.1364/OE.23.017783 26191840

[42] Tu, J., Long, K. & Saitoh, K. Graded-index few-mode multi-core fiber with dual-ring structure. *ACPC*, paper ASu4B.3, doi:10.1364/ACPC.2015.ASu4B.3 (2015).

[43] Sasaki, Y., Takenaga, K., Matsuo, S., Saitoh, K. & Koshiba, M. Trench-assisted low-crosstalk few-mode multicore fiber. *ECOC*, paper Mo.3.A.5, doi:10.1049/cp.2013.1276 (2013).

[44] Arriola A (2013). Low bend loss waveguides enable compact, efficient 3D photonic chips. Opt. Express.

[45] Thornburg W, Corrado B, Zhu X (1994). Selective launching of higher-order modes into an optical fiber with an optical phase shifter. Opt. Lett..

[46] Fontaine NK, Ryf R, Bland-Hawthorn J, Leon-Saval SG (2012). Geometric requirements for photonic lanterns in space division multiplexing. Opt. Express.

[47] Liu, B., Zhang, L. & Xin, X. A novel MIMO DSP based on matrix transformation for joint few-mode/multi-core optical transmission system. *ACPC*, paper ASu5D.4, doi:10.1364/ACPC.2015.ASu5D.4 (2015).

[48] Winzer PJ, Foschini GJ (2011). MIMO capacities and outage probabilities in spatially multiplexed optical transport systems. Opt. Express.

[49] Bai N (2012). Mode-division multiplexed transmission with inline few-mode fiber amplifier. Opt. Express.

[50] Huang B (2015). All-fiber mode-group-selective photonic lantern using graded-index multimode fibers. Opt. Express.

[51] Gross, S., Riesen, N., Love, J. & Withford, M. C-band mode-selective couplers fabricated by the femtosecond laser direct-write technique. *OFC*, paper W3B.2, doi:10.1364/OFC.2015.W3B.2 (2015).

[52] Chen H (2016). Integrated cladding-pumped multicore few-mode erbium-doped fibre amplifier for space-division-multiplexed communications. Nat. Photonics.

